# Quality of life of children with spinal muscular atrophy and their caregivers from the perspective of caregivers: a Chinese cross-sectional study

**DOI:** 10.1186/s13023-020-01638-8

**Published:** 2021-01-06

**Authors:** Mei Yao, Ying Ma, Ruiying Qian, Yu Xia, Changzheng Yuan, Guannan Bai, Shanshan Mao

**Affiliations:** 1 Department of Neurology, Children’s Hospital, Zhejiang University School of Medicine, National Clinical Research Center for Child Health, Hangzhou, 310052 China; 2 School Public Health of Zhejiang University, Children’s Hospital, Zhejiang University School of Medicine, National Clinical Research Center for Child Health, Hangzhou, 310052 China

**Keywords:** Spinal muscular atrophy, Quality of life, Disease-related characteristic, Medical intervention, Proxy-report

## Abstract

**Background:**

Spinal muscular atrophy (SMA) is an autosomal-recessive motor neuron disease leading to dysfunction of multiple organs. SMA can impair the quality of life (QoL) of patients and family. We aimed to evaluate the QoL of children with SMA and their caregivers and to identify the factors associated with QoL in a cross-sectional study conducted in China.

**Methods:**

We recruited 101 children aged 0–17 years with SMA and their caregivers from a children’s hospital in China. Twenty-six children had type I SMA, 56 type II and 19 type III. Each child’s QoL was measured by the Pediatric Quality of Life Inventory 3.0 Neuromuscular Module (PedsQL NMM), which was completed by the child’s caregivers. The caregiver’s QoL was measured by the Pediatric Quality of Life Inventory Family Impact Module (PedsQL FIM). Information on sociodemographic characteristics, disease-specific characteristics, and treatments were collected using the proxy-reported questionnaire. Two-sample *t* tests and one-way ANOVA were used to compare differences in average scores of QoL across subgroups.

**Results:**

Children with type III SMA had a higher average Total score of PedsQL NMM and higher average scores in domains Neuromuscular disease and Family resources than children with type I or type II SMA (*p* < 0.001). Caregivers of children with type III SMA reported higher average scores in the domains of Physical, Emotional, Social, and Cognitive functioning of the PedsQL FIM than those of children with types I or II SMA (*p* < 0.05). In addition, disease-related characteristics (e.g. limited mobility, stable course of disease, skeleton deformity, and digestive system dysfunction) and respiratory support were associated with lower average scores of PedsQL NMM and PedsQL FIM (*p* < 0.05). Exercise training, multidisciplinary team management and use of the medication Nusinersen were each associated with higher average scores in both PedsQL NMM and FIM (*p* < 0.05).

**Conclusion:**

Our study has demonstrated factors that may impair or improve QoL of children patients with SMA and their parents. Particularly, QoL was relatively poor in children with type I and type II SMA as well as in their caregivers compared to those with type III SMA. We strongly recommend that standard of care in a multidisciplinary team be strengthened to improve the QoL of SMA patients. Our study called for increased attention from clinical physicians on measuring QoL in their clinical practices in order to enhance the understanding of impacts of SMA and to make better decisions regarding treatment.

## Introduction

Spinal muscular atrophy (SMA) is a rare, autosomal-recessive neuromuscular disease caused by genetic mutation of the survival motor neuron (SMN) 1 gene on chromosome 5q13. This mutation results in reduced levels of the SMN protein, causing muscle weakness and atrophy [1–5]. SMA is traditionally divided into five clinical subtypes (type 0, I, II, III, IV) based on the age of onset of symptoms and on the highest achieved motor-function milestones [6, 7]. Type 0 is the most severe subtype that onsets during the prenatal period and survives less than one month after birth. Type I is the most common subtype in living patients; it usually presents before 6 months of age. Non-sitter always refer to type I patients who were never learn to sit up independently, and their life expectancy is seldom more than 2 years without respiratory support. Type II usually onsets between 6 and 18 months of age. Sitter was defined as who can sit independently but are never be able to walk. Type II patients usually can live to adulthood. Symptoms of type III normally present after age 18 months; walker refer to some patients can acquire independent ambulation, although some may lose the ability to walk in adulthood owing to the progressive nature of the disease. Life spans of these patients are almost identical to those of the general population. Type IV is the rarest, has the lowest morbidity and mortality, and occurs after 20 years of age. Life span of type IV patients is similar to that of type III patients [8, 9].

In addition to causing damage of motor neurons, SMN protein deficiency is also detrimental to the functioning of multiple tissues including those found in skeletal muscle, heart, autonomic and enteric nervous systems, metabolic/endocrine system, lymphatic systems, and reproductive system [10–12]. Therefore, dysfunction of multiple organ systems may occur in a SMA patient as the disease progresses. SMA patients may need medical care and nursing support for daily activities as well as for long-term co-management of several medical devices [13, 14]. Current treatment options in SMA include (1) ventilatory support including non-invasive and invasive ventilation, (2) management of secretions including inhalational therapies and section, (3) feeding support including nasal feeding or placement of a percutaneous endoscopid gastrostomy and (4) supply with medical devices and orthosis [15–17]. Improvements regarding survival and QoL of SMA patients could be achieved by these options [7].

QoL is a multidimensional concept and is an important patient-reported outcome measure in clinical research and practice. According to the World Health Organization, QoL is defined as “an individual’s perception of their position in the life in the context of the culture in which they live and in relation to their goals, expectations, standards and concerns” [18]. While investigating QoL in the clinical setting, it is important to understand the patient’s experience, to evaluate the effectiveness of treatments, and to optimize individual therapy plans, which ultimately improve the patient’s well-being [19, 20].

In pediatric neurology research, attentions mostly has focused on developing novel technologies and pharmaceuticals for children with SMA. Few studies have focused on investigating the impacts of these clinical treatments on the QoL of patients and caregivers. One study conducted in Europe by Rouault et al. described the disease impact on general well-being and therapeutic expectations of SMA type II and III patients [21]. This study highlighted the patient’s perspective in terms of treatment and living with SMA [21]. However, the study used a self-developed questionnaire based on expert opinion instead of a validated survey instrument for measuring QoL. Based on a literature review, we found that the PedsQL 3.0 Neuromuscular Module (PedsQL NMM) can be used to estimate the QoL of SMA patients, whereas the PedsQL Family Impact Module (PedsQL FIM) can be used as a supplement of the PedsQL NMM to reflect the caregivers’ QoL and the family impact. Weaver et al. found that PedQL FIM captured significant differences in functioning domains of QoL including physical, emotional, social, and family relations between SMA Type I and II [22]. In addition, this study demonstrated significant differences in the communication domain of the proxy-reported PedsQL NMM [22]. Nusinersen treatment did not impact proxy-reported QoL, whereas gastrostomy tube and ventilation support decreased children’s QoL [22]. The analysis of parents' questionnaires revealed that different types of SMA and clinical treatment can significantly affect the QoL of SMA patients. Published studies of the QoL of SMA patients and caregivers have been conducted in the United States or in Europe. To our best knowledge, no similar study has been conducted in China. Therefore, in the present study we aimed to evaluate the QoL of children with SMA and their caregivers and to identify the factors associated with QoL (such as SMA subtypes, disease-related characteristics and treatments) for patients and caregivers that participated in a hospital-based, cross-sectional study in China.

## Methods

### Participants and design

During 16–28 March 2020 we conducted a questionnaire-based survey of 101 caregivers, which included the parents or grandparents of SMA patients recruited from the Department of Neurology of the Children's Hospital of Zhejiang University School of Medicine in Hangzhou, China. The inclusion criteria for caregivers were (1) their child was diagnosed as SMA by genetic testing and (2) their child’s age ranged from 0 to 18 years. Caregivers were excluded if they could not understand or complete the questionnaire correctly. In accordance with the principle of voluntary participation, the study questionnaire was sent by website to the caregivers with detailed information about the purpose and methods of this study. All eligible participants provided signed, informed consent before they completed the questionnaire, and the study was approved by the Ethics Committee of the Children’s Hospital of Zhejiang University School of Medicine (2019-IRB-171).

Before the questionnaire was used, we conducted a preliminary test of the feasibility, comprehensibility, and acceptability of the questions (survey items) and their multiple-choice answers. During this test 25 questionnaires were sent out, 24 were received yielding an effective recovery of 96%. The results of the preliminary test showed that the population response rate was very high and that our investigation was feasible. It took the caregiver 20–30 min to fill in the questionnaire, so we set the recommended length of time of completion of questionnaire as 35 min in the instruction texts of the final survey questionnaire. During the survey of all participants, the data were collected by trained physicians using a one-to-one internet survey. After logging onto the questionnaire website, all caregivers were asked to complete the PedsQL FIM section. In addition, the caregivers of SMA patients aged 5 years and older were asked to complete the PedsQL NMM section. All caregivers immediately submitted their answers upon completion of the questionnaire.

### QoL measures

PedsQL NMM and PedsQL FIM were used to measure QoL of patients with SMA and their caregivers. In addition, the questionnaire included some questions to collect the information on socio-demographic characteristics, disease-related characteristics, and medical interventions/treatment plan of the patients. Several terms were used in these additional questions. For example, a non-Sitter was defined as a patient who was unable to sit. A sitter was defined as a patient who could sit without aid but could not walk alone, whereas a patient who could walk alone was defined as walker. Skeletal dysfunctions were defined as structural changes in the skeletal system that result in functional changes, such as scoliosis, hip dislocation, tendon contracture, joint deformation, etc. Digestive system dysfunctions were defined as disordered functions of digestive organs including the gastrointestinal tract, the liver and biliary system, and the pancreas. The main symptoms of digestive system dysfunctions included salivation, difficulty swallowing, constipation, etc. Stable course of disease was defined as a state of disease progression wherein patient’s athletic ability and intensity keep no progressive levels during past 6 months. For example, a patient who was 9 months old but was unable to hold her/his head up steadily from 3 months old. Exercise training was defined as rehabilitation training in professional institutions. A patient that had not accepted any medical service was defined as no-medical service (N-MS). A patient that only accepted a neurology doctor but was not under multidisciplinary team (MDT) was defined as medical service (MS). A patient that accepted MDT was defined as accepted standard of care (SOC) in a MDT management program.

We used PedsQL NMM, a proxy-reported, 25-item survey instrument, to measure health-related QoL in children with neuromuscular disorders. This instrument contains three dimensions: About My Child’s Neuromuscular Disease (17 items, with emphasis on physical functioning), Communication (three items), and About Our Family Resources (five items). For each item, caregivers were asked to rate the influence of a certain problem in the past month. Each multiple-choice answer was scored from 0 (never a problem) to 4 (almost always a problem). Item scores were reversed and linearly transformed to a 0 to 100 scale (0 = 100, 1 = 75, 2 = 50, 3 = 25, and 4 = 0); therefore, a higher scores indicated a higher QoL. PedsQL NMN is a reliable survey instrument, and Cronbach’s alpha for each dimension exceeds 0.70 [23].

The PedsQL FIM was designed to assess the impact of pediatric chronic health conditions on caregivers’ QoL and family functions in the past month. The PedsQL FIM includes the following six scales of a caregiver’s self-reported functioning: physical functioning (six items), emotional functioning (five items), social functioning (four items), cognitive functioning (five items), communication (three items), and worry (five items). In addition, the PedsQL FIM explored the following two scales of family functioning reported by the primary caregiver: daily activities (three items) and family relationships (five items). Each item included a five-point, multiple-choice response ranging from 0 (never a problem) to 4 (always a problem). These item scores were reversely graded and linearly transformed to a 0–100 scale (0 = 100, 1 = 75, 2 = 50, 3 = 25, 4 = 0); therefore, higher scores indicated better functioning or less negative impacts. Initial validation studies indicated that PedsQL FIM has a relatively high internal consistency with Cronbach’s alpha values exceeding 0.70 [24].

### Statistical analysis

Descriptive analysis was applied to describe the characteristics of the study population. Continuous measurements were summarized as means ± standard deviations (SDs), and categorical measurements were summarized using frequencies with percentages. A normal distribution of our data was tested using the normality test and distribution curve. A two-sample t-test was used to compare the difference in average scores of QoL between two subgroups; whereas a one-way ANOVA was used to compare average scores of QoL among three or more subgroups. In addition, we used Cohen’s effect size (Cohen’s *d*) to assess the clinical relevance in terms of pairwise differences in average score between groups (including differences between subtypes of SMA, disease-related characteristics, and acceptance categories of clinical treatments). Cohen’s *d* was calculated as the absolute value of the difference in average scores divided by the largest SD and was interpreted as follows: 0.2 ≤ *d* < 0.5, small difference; 0.5 ≤ *d* < 0.8, moderate difference; and *d* ≥ 0.8, large difference [25].

All statistical tests were two-tailed and their significance was indicated by *p* < 0.05. All the analyses were conducted using SPSS version 22 (IBM Corp. NY, USA).

## Results

### Characteristics of the study population

Socio-demographics characteristics, disease-related characteristics and medical interventions/treatment plan of the patients in our study are summarized in Table 1. A total of 101 caregivers with SMA children participated in our study. All caregivers completed the PedsQL FIM questionnaire; however, only 87 caregivers completed the PedsQL NMM questionnaire because this survey instrument applies only to children of ages 2–18 years. Ten patients depended on invasive ventilation, 23 patients used a suction tube and only 1 child received a non-invasive ventilator treatment. Sixteen patients were treated by special formula feeding, 2 patients used nasal feeding tube and 1 child received gastrostomy in nutritional support.

**Table 1:** The demographics characteristics of SMA patients (n = 101)

Variable	*N* (%)
Child sex	
Male	49 (48.50)
Female	52 (51.50)
Child primary diagnosis	
SMA type I	26 (25.70)
SMA type II	56 (55.40)
SMA type III	19 (18.80)
Average age of SMA type (years)	Age range of SMA type (years)
SMA type I: 5.28	0.50–16.17
SMA type II: 6.90	0.92–16.00
SMA type III: 9.35	2.08–13.67
Disease duration	Age range of disease duration (years)
SMA type I	4.86 (0.33–15.58)
SMA type II	6.12 (0.17–15.50)
SMA type III	5.9 (1.00–13.67)
Mobility	
Non-sitter	46 (45.50)
Sitter	39 (38.60)
Walker	16 (15.80)
Stable course of disease	
No	41 (40.60)
Yes	60 (59.40)
Skeletal deformity	
No	27 (26.70)
Yes	74 (73.30)
Digestive system dysfunction	
No	78 (77.20)
Yes	23 (22.80)
Medical services	
N-MS	67 (66.30)
MS but not U-MDT	21 (20.80)
U-MDT	13 (12.90)
Exercise training	
No	56 (55.40)
Yes	45 (44.60)
Respiratory support	
No	75 (74.30)
Yes	26 (25.70)
Nutritional support	
No	82 (81.20)
Yes	19 (18.80)
Scoliosis surgery	
No	99 (98.00)
Yes	2 (2.00)
Nusinersen treatment	
No	92 (91.10)
Yes	9 (8.90)

*SMA* spinal muscular atrophy, *N-MS* no medical services, *MS but not, U-MDT* medical services but not under multidisciplinary team, *U-MDT* Under multidisciplinary team

### Differences in the average scores of PedsQL NMM across SMA subtypes

The average scores of PedsQL NMM stratified by SMA subtypes are summarized in Table 2. The average total score and average scores of domains Neuromuscular diseases and Family resources were significantly higher in patients with SMA type III than in those with SMA type I (64.89 vs. 43.63, *p* < 0.001, *d* = 1.08; 65.32 vs. 40.53, *p* < 0.001, *d* = 1.23; and 58.68 vs. 38.75, *p* = 0.006, *d* = 0.74). Similarly, the average total score and average scores of domains Neuromuscular diseases and Family resources were significantly higher in patients with SMA type III than in those with SMA type II (64.89 vs. 48.79, *p* < 0.001, *d* = 0.82; 65.32 vs. 49.10, *p* < 0.001, and 58.68 vs. 33.94, *p* < 0.001, *d* = 0.92). In contrast, no statistically significant differences were found among SMA subtypes in the average score of domain Communication.

**Table 2:** Average scores of proxy-reported PedsQL 3.0 Neuromuscular Module across SMA subtypes (n = 87)

Children’s QoL accessed from the caregiver	SMA Type I Mean ± SD (*n* = 16)	SMA Type II Mean ± SD (n = 52)	SMA Type III Mean ± SD (*n* = 19)	Effect size (d)		
				I versus II	I versus III	II versus III
Total score	**43.63 ± 13.08**	**48.79 ± 13.04**	**64.89 ± 19.75**^**b,c**^	0.39	1.08	0.82
Neuromuscular disease	**40.53 ± 13.86**	**49.10 ± 13.65**	**65.32 ± 20.21**^**b,c**^	0.62	1.23	0.80
Communication	69.27 ± 33.85	71.79 ± 26.72	74.56 ± 27.70	0.07	0.16	0.10
Family resources	**38.75 ± 13.60**	**33.94 ± 20.28**	**58.68 ± 26.87**^**b,c**^	0.24	0.74	0.92

Effect size (Cohen’s *d*) is interpreted as: 0.2 ≤ *d* < 0.5 small difference, 0.5 ≤ *d* < 0.8 moderate, and *d* ≥ 0.8 large

*SMA* spinal muscular atrophy

Bold prints indicate *P* < 0.05

^a^Post hoc significance between type I and type II

^b^Post hoc significance between type I and type III

^c^Post hoc significance between type II and type III

The average scores of PedsQL FIM stratified by subtype are summarized in Table 3. The average total score and the average scores of domains Physical, Emotional, Social, Cognitive functioning were significantly higher in patients with SMA type III than in those with SMA types I or II (*p* < 0.05). The largest effect size was seen in the average scores of domain Physical functioning when comparing patients of SMA types III and I (*d* = 1.04). No statistically significant differences were found in the above-mentioned average scores between patients of SMA types I and II (*p* > 0.05).

**Table 3:** Average scores of PedsQL Family Impact Module across SMA subtypes (n = 101)

Caregivers’ quality of life	SMA Type I Mean ± SD (*n* = 26)	SMA Type II Mean ± SD (*n* = 56)	SMA Type III Mean ± SD (*n* = 19)	Effect size (d)		
				I versus II	I versus III	II versus III
Total score	**34.97 ± 21.97**	**40.56 ± 17.76**	**53.80 ± 21.87**^**b,c**^	0.25	0.86	1.45
Physical functioning	**29.01 ± 25.18**	**45.16 ± 21.52**	**59.43 ± 29.23**^**a,**^^**b,c**^	0.64	1.04	0.49
Emotional functioning	**31.54 ± 28.42**	**33.93 ± 20.64**	**54.47 ± 23.27**^**b,c**^	0.08	0.81	0.88
Social functioning	**29.33 ± 26.15**	**37.28 ± 25.11**	**55.59 ± 29.16**^**b,c**^	0.30	0.90	0.63
Cognitive functioning	**40.00 ± 24.78**	**45.18 ± 24.77**	**61.58 ± 25.71**^**b,c**^	0.21	0.84	0.64
Communication	41.99 ± 31.49	43.75 ± 25.69	55.26 ± 31.21	0.06	0.42	0.37
Worry	30.00 ± 26.87	26.16 ± 17.94	37.63 ± 27.35	0.14	0.28	0.42
Daily activities	28.20 ± 25.94	36.61 ± 24.40	40.79 ± 24.52	0.32	0.49	0.17
Family relationships	49.81 ± 28.02	54.46 ± 21.78	60.26 ± 23.24	0.17	0.37	0.25

Effect size (Cohen’s *d*) is interpreted as: 0.2 ≤ *d* < 0.5 small difference, 0.5 ≤ *d* < 0.8 moderate, and *d* ≥ 0.8 large

*SMA* spinal muscular atrophy

Bold prints indicate *P* < 0.05

^a^Post hoc significance between type I and type II

^b^Post hoc significance between type I and type III

^c^Post hoc significance between type II and type III

### Differences in the average scores of PedsQL NMM across disease-specific characteristics

The average score of PedsQL NMM stratified by disease-related characteristics are summarized in Table 4. Patients categorized as non-sitter or sitter were reported by their parents to have relatively average scores (Total and domains Neuromuscular disease and Family resources) compared to parents that were able to walk independently (*p* < 0.05). In particular, in comparisons of Non-sitter or Walker, the effect sizes were 1.19, 1.35 and 1.07 for differences in average Total score, average score of domain Neuromuscular disease, and average score of domain Family resources, respectively. The average Total score and the average scores of domains Neuromuscular disease and Family resources were significantly higher in children without stable course of disease than in those with stable course of disease (*p* < 0.01); the effect sizes ranged from 0.29 to 0.64. The average Total scores and the average scores of domains Neuromuscular disease and Family resources were significantly higher in children without motor skeleton deformity than in those with skeleton deformity (*p* < 0.05); the effect sizes ranged from 0.46 to 0.49. The average Total scores and the average scores of domains Neuromuscular disease and Family resources were significantly higher among children with normal digestive system function than in those with digestive system dysfunction (*p* < 0.01); the effect sizes ranged from 0.68 to 1.06.

**Table 4:** Average scores of proxy-reported PedsQL 3.0 Neuromuscular Module across disease-related characteristics (n = 87)

	Mobility						Stable course of disease			Skeletal deformity			Digestive system dysfunction		
	Non-sitter (n = 34)	Sitter (n = 37)	Walker (n = 16)	Effect size (d)			No (n = 33)	Yes (n = 54)	Effect size(d)	No (n = 21)	Yes (n = 66)	Effect size(d)	No (n = 70)	Yes (n = 17)	Effect size(d)
				Non-sitter versus sitter	Non-sitter versus Walker	Sitter versus Walker									
Total score	**43.03 ± 11.98**	**52.51 ± 13.48**^**a**^	**66.38 ± 19.58**^**b,c**^	0.70	1.19	0.71	**57.67 ± 15.51**	**47.50 ± 15.80**	0.64	**57.67 ± 17.84**	**49.35 ± 15.48**	0.47	**54.20 ± 16.28**	**39.65 ± 10.77**	0.89
Neuromuscular disease	**40.27 ± 12.70**	**53.97 ± 12.68**^**a**^	**67.28 ± 19.98**^**b,c**^	1.08	1.35	0.67	**57.53 ± 16.46**	**47.11 ± 16.65**	0.63	**57.56 ± 17.31**	**49.00 ± 16.83**	0.49	**54.52 ± 16.68**	**36.85 ± 11.48**	1.06
Communication	72.79 ± 28.89	69.82 ± 26.89	75.00 ± 30.28	0.10	0.07	0.17	76.77 ± 27.54	68.98 ± 28.20	0.28	73.41 ± 26.83	71.46 ± 28.61	0.07	71.07 ± 28.51	75.49 ± 26.59	0.16
Family resources	**34.56 ± 16.67**	**37.16 ± 23.99**	**59.38 ± 23.23**^**b,c**^	0.11	1.07	0.93	**46.67 ± 24.20**	**36.30 ± 21.44**	0.29	**49.52 ± 26.64**	**37.27 ± 21.02**	0.46	**43.29 ± 23.14**	**27.65 ± 17.69**	0.68

Values in the table are means, standard deviations and effect sized. Effect size (Cohen’s *d*) is interpreted as: 0.2 ≤ *d* < 0.5 small difference, 0.5 ≤ *d* < 0.8 moderate, and *d* ≥ 0.8 large

Bold prints indicate *P* < 0.05

^a^Post hoc significance between non-sitter and sitter

^b^Post hoc significance between non-sitter and walker

^c^Post hoc significance between sitter and walker

### Differences in the average scores of PedsQL FIM across disease-specific characteristics

The average scores of PedsQL FIM stratified by disease-related characteristics are summarized in Table 5. Parents with children categorized as Non-sitter or Sitter reported significantly lower average Total scores and average scores of domains Physical, Emotional, Social, Cognitive functioning compared with the same scores of children categorized as Walker *(p* < 0.05); the largest effect size was 1.40 in the comparison between Non-sitter and Walker categories. The average scores of domains Physical and Emotional functioning were significantly lower for children with skeletal deformity than for those without skeletal deformity (*p* < 0.05). Regarding digestive system dysfunction, the average Total score and the average scores of domain Daily activities and domains Physical, Emotional, Social, Cognitive functioning were significantly lower in children with the dysfunction than in those that lacked the dysfunction (*p* < 0.01); the effect sizes ranged from 0.56–1.26.

**Table 5:** Average scores of PedsQL Family Impact Module across disease-related characteristics (n = 87)

Caregivers’ QoL	Mobility Mean ± SD						Skeletal deformity Mean ± SD			Digestive system dysfunction Mean ± SD		
	Non-Sitter (n = 46)	Sitter (n = 39)	Walker (n = 16)	Effect size (d)			No (n = 27)	Yes (n = 74)	Effect size (d)	No (n = 78)	Yes (n = 23)	Effect size (d)
				Non-sitter versus Sitter	Non-sitter versus Walker	Sitter versus walker						
Total score	**50.43 ± 24.33**	**56.28 ± 24.03**	**60.94 ± 20.83**^**b,c**^	0.24	0.43	0.19	47.94 ± 24.78	39.29 ± 18.37	0.35	**45.05 ± 19.63**	**29.92 ± 19.45**	0.77
Physical functioning	**34.33 ± 24.89**	**45.30 ± 23.14**^**a**^	**66.67 ± 20.53**^**b,c**^	0.44	1.40	0.86	**55.86 ± 23.58**	**39.25 ± 25.43**	0.65	**50.37 ± 22.77**	**21.01 ± 23.25**	1.26
Emotional functioning	**31.30 ± 25.96**	**36.92 ± 21.29**	**54.69 ± 20.69**^**b,c**^	0.22	1.10	0.68	**46.67 ± 26.67**	**33.72 ± 23.00**	0.49	**41.15 ± 22.53**	**23.70 ± 26.94**	0.65
Social functioning	**32.34 ± 26.46**	**37.34 ± 24.81**	**60.16 ± 26.70**^**b,c**^	0.19	1.04	0.85	46.07 ± 35.45	35.98 ± 23.47	0.28	**42.15 ± 27.09**	**26.90 ± 25.45**	0.56
Cognitive functioning	**42.07 ± 23.13**	**46.03 ± 26.39**	**63.13 ± 26.64**^**b,c**^	0.15	0.79	0.64	53.89 ± 29.40	44.40 ± 24.06	0.32	**51.22 ± 25.37**	**32.39 ± 22.00**	0.74
Communication	43.48 ± 29.91	42.31 ± 24.29	58.85 ± 31.40	0.04	0.49	0.53	48.15 ± 33.60	44.48 ± 26.50	0.11	47.87 ± 27.01	37.32 ± 32.16	0.33
Worry	29.57 ± 22.85	24.62 ± 18.79	40.00 ± 27.69	0.22	0.38	0.56	29.81 ± 29.92	29.12 ± 19.52	0.02	29.74 ± 22.75	27.83 ± 22.55	0.08
Daily activities	32.43 ± 24.48	35.04 ± 25.30	43.75 ± 25.18	0.10	0.45	0.34	36.42 ± 30.24	34.79 ± 22.97	0.05	**38.35 ± 25.35**	**24.64 ± 20.79**	0.54
Family relationships	50.43 ± 24.33	56.28 ± 24.03	60.94 ± 20.83	0.2	0.44	0.19	60.18 ± 25.02	52.23 ± 23.15	0.32	56.35 ± 22.94	47.61 ± 25.89	0.34

Values in the table are means, standard deviations and effect sized. Effect size (Cohen’s *d*) is interpreted as: 0.2 ≤ *d* < 0.5 small difference, 0.5 ≤ *d* < 0.8 moderate, and *d* ≥ 0.8 large

Bold prints indicate *P* < 0.05

^a^Post hoc significance between non-sitter and sitter

^b^Post hoc significance between non-sitter and walker

^c^Post hoc significance between sitter and walker

### Differences in the average scores of PedsQL NMM across the clinical treatments

The average scores of PedsQL NMM stratified by clinical treatments are summarized in Table 6. The average Total scores and the average scores of domain Neuromuscular disease were significant higher in children that received Exercise training than in those which did not (*p* < 0.001); the effect sizes were 0.60 and 0.75, respectively. The average Total scores and the average scores of domain Neuromuscular disease were significant lower in children that received Respiratory support than in those which did not (*p* < 0.001); the effect sizes were 0.57 and 0.79, respectively. Regarding MDT, significant differences in average Total score and in average scores of domains Neuromuscular disease and Family resources were detected between groups with SOC in MDT and groups without MDT (N-MS and MS groups). Children in the MS group had significantly higher average Total scores and average scores in domains Neuromuscular disease and Family resources than children in N-MS group (*p* < 0.05). Children under MDT had significantly higher average Total scores and average scores in domains Neuromuscular disease and Family resources than children in N-MS groups (*p* < 0.05) or those in MS group (*p* < 0.05). Effect sizes ranged from 0.13 to 0.90.

**Table 6:** Average scores of proxy-reported PedsQL 3.0 Neuromuscular Module across subgroups of clinical treatments (n = 87)

	Exercise training			Respiratory support			MDT					
	No (*n* = 49)	Yes (*n* = 38)	Effect size *(d)*	No (*n* = 64)	Yes (*n* = 23)	Effect size *(d)*	N-MS (*n* = 58)	MS but not U-MDT (*n* = 16)	U-MDT (*n* = 13)	Effect size*(d)*		
										N-MS versus MS but not U-MDT	N-MS versus U-MDT	MS but not U-MDT versus U-MDT
Total score	**46.51 ± 12.83**	**57.61 ± 18.39**	0.60	**53.84 ± 16.62**	**44.43 ± 13.72**	0.57	**47.26 ± 13.45**	**60.44 ± 19.48**^**a**^	**58.46 ± 18.46**^**b,c**^	0.68	0.61	0.10
Neuromuscular disease	**45.38 ± 14.98**	**58.40 ± 17.38**	0.75	**54.62 ± 17.00**	**41.18 ± 14.02**	0.79	**47.26 ± 14.66**	**60.39 ± 20.11**^**a**^	56.56 ± 19.67	0.65	0.47	0.19
Communication	71.09 ± 26.58	73.03 ± 30.17	0.06	72.14 ± 28.10	71.38 ± 28.52	0.03	71.41 ± 27.00	68.23 ± 35.12	78.85 ± 23.72	0.09	0.28	0.30
Family resources	36.02 ± 17.02	45.66 ± 28.19	0.34	40.55 ± 23.45	39.35 ± 21.97	0.05	**33.10 ± 18.23**	**55.94 ± 25.44**^**a**^	**52.69 ± 25.38**^**b,c**^	0.90	0.77	0.13

Effect size (Cohen’s *d*) is interpreted as: 0.2 ≤ *d* < 0.5 small difference, 0.5 ≤ *d* < 0.8 moderate, and *d* ≥ 0.8 large

*N-MS* no medical services, *U-MDT* under multidisciplinary team, *MS but not U-MDT* medical services but not under multidisciplinary team

Values in the table are means, standard deviations and effect sized. Bold prints indicate *P* < 0.05

^a^Post hoc significance between no medical services and medical services but not under MDT

^b^Post hoc significance between no medical services and under MDT

^c^Post hoc significance between medical services but not under MDT and under MDT

## Discussion

The present study assessed proxy-reported quality of life of children with SMA and self-reported quality of life of their caregivers in China. We found that quality of life of patients and caregivers differed across SMA subtype, disease-related characteristics, and treatment characteristics.

We found average scores were higher in terms of the overall quality of life score, Neuromuscular diseases domain, and Family resource domain of PedsQL NMM of patients with type III compared with those in patients with type II or type I, which was consistent with previous studyhaving the lowest average score. It could be explained by the difference in severity across subtypes. Additionally, the effect sizes were moderate to large, which indicated that the differences in quality of life of patients had reached the threshold of clinical relevance and were worthy of attention from health professionals. Meaghann et al*.* found no statistically significant difference across SMA subtypes in the Neuromuscular diseases domain of PedsQL NMM, which was different with our finding [22]. It might be explained by the difference in the severity of SMA between our sample and theirs. Patient’s physical function was worse in our sample, especially in patients with type I and II.

Our study showed better QoL of caregivers of patients with type III than those of patients with type I and II, which was explained by the nature of the three subtypes of SMA. Type III patients were the least severe type. Most patients with type III have the ability to move autonomously, to complete various functional exercises by themselves, and are able to walk alone, which may put less burden on their caregivers, compared with other two types of patients who cannot walk or sit. Our study did not find significant difference across SMA types in the Communication, Worry, Daily activities, or Family relations domains of PedsQL FIM. The potential explanation was that regardless of subtypes, all the caregivers gradually accepted the fact of living together with ill children and adapted to the situation. So the impact of SMA on caregiver’s worry level, daily activities and on family communication about patients/disease and family relations may stay stable during treatment phase. That may be the reason why our study did not find the differences in the above-mentioned domains across subtypes.

Disease-related characteristics of patients with SMA include skeletal malformation, digestive dysfunction, stable course of disease, and current motor ability. In the present study, the above-mentioned characteristics of SMA were associated with lower average score in Neuromuscular diseases domain and Family resources domain of PedsQL NMM than patients without these complications. As SMA is a progressive disease, complications such as skeletal malformation, digestive disorders, respiratory disorders, and motor function degeneration may influence patient’s QoL gradually [10, 11]. Our study also supported the findings from a previous study that showed motor function affected multiple aspects of the patient’s life [27]. In addition to the statistical significances, the effect sizes regarding Neuromuscular disease and Family resource domain of PedsQL NMM were moderate and large, indicating that disease-related characteristics may impact the QoL of patients, which reached the threshold of clinical relevance and warranted attention from clinical professionals during their practice.

Regarding caregiver’s QoL measured by PedsQL FIM, the physical and emotional functions of the caregivers of patients with skeletal malformations were worse compared with caregivers of patients without skeletal malformations. Skeletal malformation usually accompanies with progressive muscular atrophy and muscle weakness that often worsen skeletal malformation in return, which is a circle leading to impaired motor ability overtime [13, 14, 28]. Under this situation, caregivers had to spend lots of time and energy on daily care of patients and had fewer time for themselves, which may lead to great burden on caregiver’s physical and mental health. Our study also suggested that digestive system dysfunction may negatively influence caregiver’s QoL in multiple domains, such as physical, mental, social and cognitive function. Digestive system dysfunction such as dysphagia and other digestive disorders often appear in the last stage of SMA, which seriously affects the patient’s overall health [29]. Meanwhile, caregivers were under great burden to take care of patients, such as financial burden, less personal time and great mental stress, which deteriorated their health and QoL [17].

Our results suggested that clinical treatments may improve the QoL of patients with SMA. Early exercise training is beneficial for remaining or improving the mobile function [30, 31]. Early studies showed that exercise training can activate the motor neurons to promote the recovery, and effectively improve the motor function of patients with neuromuscular degeneration disease, including Duchenne muscular dystrophy and SMA, which eventually improved patient’s QoL [32–35].

Though previous studies showed that respiratory support could improve patient’s health outcomes [22, 36, 37], our study showed that patients with respiratory support had lower average scores and lower score of Neuromuscular disease domain of PedsQL NMM reported by caregivers, compared with those without respiratory support. The reasonable explanation was that patients received respiratory support when their condition deteriorated to a very severe situation. In this case, their overall health status and well-being actually were worse than those who did not receive respiratory support.

Our study showed that the multidisciplinary team (MDT) management was associated with better overall QoL patients compared with patients without MDT management. The effect size was moderate, which indicated a minimally clinically important difference. MDT management refers to having more than two related disciplines in order to implement clinical treatments for specific diseases [38, 39]. As shown in previous studies, MDT management in other chronic diseases, such as tuberous sclerosis complex (TSC), Prader-Willi syndrome, pediatric chronic pancreatitis, pediatric medulloblastoma, and asthma, this approach was conducive to reducing caregiver burden, to decreasing healthcare load and to improve patient’s QoL [40–43]. According to our clinical experience, low awareness of MDT health management interventions among caregivers, which may delay early interventions to SMA. Based on our observations and previous studies, we suggested that raise the caregivers’ awareness of MDT management, which is beneficial to conduct the early intervention and eventually improve the health outcomes, such as QoL of patients [13, 14].

We did not show the results in tables regarding the differences of quality of life across the subgroup using Nusinersen and not using this medication because only six patients in our study received Nusinersen treatment. Briefly, the result showed that patients with Nusinersen treatment was reported higher average score in the Neuromuscular diseases and Family resources domains of PedsQL NMM than those without such treatment. Nusinersen is the first effective medication for SMA treatment available in China. It can improve the motor, respiratory, digestive, and other system functions in patients [44–47]. Because the sample size of patients with Nusinersen treatment was very small, we suggested to interpret our finding with caution and we recommended further studies to confirm or reject our finding.

We observed that the QoL scores in our study were lower than those reported in previous studies conducted in developed country yy[22]. The potential explanations were: First, China is a developing country while American/European countries are developed ones. The cost of treatment is prohibitive for most SMA patients in China, and they cannot receive the early diagnosis and treatments obtained by patients of foreign countries. Second, the development of diagnosis and treatment of SMA in China is slower than that in foreign countries, and drugs appear on the market later than in western countries. Third, the United States and Belgium have incorporated SMA into newborn screening and has attached great importance to the diagnosis and treatment of SMA; whereas China is still in early stages.

Our study has several strengths. To our best knowledge, it was the first study to evaluate QoL of Chinese patients with SMA and to reflect the QoL of these children according to the perceptions of the caregivers by reliable and valid instruments. Additionally, we have assessed a comprehensive set of potential associated factors of QoL, including SMA subtypes, disease-related characteristics and clinical treatments. Previous studies have not provided evidence on the QoL of patients with SMA in China, and few studies exist regarding the influence of clinical treatments on QoL of patients and their caregivers. Given that China is different from developed countries in social, economic, and medical characteristics, it is necessary and important to measure QoL of SMA and their caregivers in China, as well as the determinants of QoL.

Our study had several limitations worthy to mention. First, the sample size of this study is moderate, reflecting the rarity of the disease. We have not applied self-report in our study because the sample size would be even smaller if we only included children older than 5 years. In the future study, we recommended further studies with larger samples could apply both self-reported and proxy-reported QOL measurements among patients with SMA to get a full picture of patient’s QOL. A second limitation of this study is that patients were only sampled from central and east China. Given the lack of QoL surveys on SMA patients in other regions, the study may not be representative of SMA patients across China. Nevertheless, our results may be generalized to patients from the areas mentioned above. Third, this study was a cross-sectional study with data collected at a single time point, which precluded drawing conclusions regarding causality. Fourth, owing to less data of specific items of clinical treatments, our study cannot provide a more in-depth analysis; so future research is needed with a larger sample that includes subsets of clinical treatments. In addition, our study reflected the influence of disease-related characteristics on the QoL of children and only the initial effect of medical measures. Finally, although the PedsQL is designed to be used for all types of diseases, this survey instrument has not been specifically validated for proxy-reported use in children with SMA.

In summary, further follow-ups of these patients should be conducted and QoL indicators should be used to investigate the effectiveness of clinical treatments for childhood SMA. In addition, future studies need larger sample sizes and should pursue multicenter enrollments to involve patients with SMA from all of China to generate a database that reflects the QoL of patients across the country. To confirm the effectiveness of treatment approaches and to make the results more generalizable, subsequent studies should continue research in the diagnosis and treatment of SMA, and should include larger numbers of patients, especially in the drug treatment group.

## Conclusion

To conclude, the more severe the SMA disorder was, the lower were the average scores on the PedsQL NMM and PedsQL FIM as reported by the patients’ caregivers, and the poorer was the patients’ QoL. Disease-related clinical features and clinical treatments have a significant influence on the QoL of patients with SMA. We recommend implementing clinical treatments, supplemented by exercise training and respiratory support, regularly assessing the functional status of various systems, and establishing MDT health management in patients as early as possible, which can effectively prevent or delay the emergence of disease-related clinical characteristics, can improve patients’ QoL, and can relieve caregivers’ psychological pressures and overall life burden.

## Supplementary Information

**Additional file 1**. Detailed dimension of proxy-report of PedsQL ™Neuromuscular Module and PedsQL™ Family Impact Module.

## Data Availability

All data generated during this study are included in this published article.

## Abbreviations

SMA: Spinal muscular atrophy
QoL: Quality of life
SMN1: Survival motor neuron 1
SMN: Survival of motor neuron
PedsQL NMM: Pediatric Quality of Life Inventory 3.0 Neuromuscular Module
PedsQL FIM: Pediatric Quality of Life Inventory Family Impact Module
MDT: Multidisciplinary team
SD: Standard deviation
SOC: Standard of care
