# Supplementary material for: Quality of life of children with spinal muscular atrophy and their caregivers from the perspective of caregivers: a Chinese cross-sectional study
Source: Orphanet J Rare Dis. 2021 Jan 6;16:7. doi: 10.1186/s13023-020-01638-8 (PMC7789582; doi:10.1186/s13023-020-01638-8)
Supplement: Supplementary file 1 — Additional file 1. Detailed dimension of proxy-report of PedsQL ™Neuromuscular Module and PedsQL™ Family Impact Module. [file 13023_2020_1638_MOESM1_ESM.docx]

**Supplementary table1:**

**PedsQL ™Neuromuscular Module（proxy-report）**

*In the past ONE month, how much of a problem has this been for your child…*

| **ABOUT MY CHILD’S NEUROMUSCULAR DISEASE**  ***(problems with…)*** | **Never** | **Almost Never** | **Some- times** | **Often** | **Almost Always** |
| --- | --- | --- | --- | --- | --- |
| 1. It is hard for my child to breathe | 0 | 1 | 2 | 3 | 4 |
| 2. My child gets sick easily | 0 | 1 | 2 | 3 | 4 |
| 3. My child gets sores and/or rashes | 0 | 1 | 2 | 3 | 4 |
| 4. My child’s legs hurt | 0 | 1 | 2 | 3 | 4 |
| 5. My child feels tired | 0 | 1 | 2 | 3 | 4 |
| 6. My child’s back feels stiff | 0 | 1 | 2 | 3 | 4 |
| 7. My child wakes up tired | 0 | 1 | 2 | 3 | 4 |
| 8. My child’s hands are weak | 0 | 1 | 2 | 3 | 4 |
| 9. It is hard for my child to use the bathroom | 0 | 1 | 2 | 3 | 4 |
| 10. It is hard for my child to gain or lose weight when he or she wants to | 0 | 1 | 2 | 3 | 4 |
| 11. It is hard for my child to use his or her hands | 0 | 1 | 2 | 3 | 4 |
| 12. It is hard for my child to swallow food | 0 | 1 | 2 | 3 | 4 |
| 13. It takes my child a long time to bathe or shower | 0 | 1 | 2 | 3 | 4 |
| 14. My child gets hurt accidentally | 0 | 1 | 2 | 3 | 4 |
| 15. My child takes a long time to eat | 0 | 1 | 2 | 3 | 4 |
| 16. It is hard for my child to turn him or herself during the night | 0 | 1 | 2 | 3 | 4 |
| 17. It is hard for my child to go places with his or her equipment | 0 | 1 | 2 | 3 | 4 |
| **COMMUNICATION *(problems with…)*** | **Never** | **Almost Never** | **Some- times** | **Often** | **Almost Always** |
| 1. It is hard for my child to tell the doctors and nurses how he or she feels | 0 | 1 | 2 | 3 | 4 |
| 2. It is hard for my child to ask the doctors and nurses questions | 0 | 1 | 2 | 3 | 4 |
| 3. It is hard for my child to explain his or her illness to other people | 0 | 1 | 2 | 3 | 4 |
| **ABOUT OUR FAMILY RESOURCES *(problems with…)*** | **Never** | **Almost Never** | **Some- times** | **Often** | **Almost Always** |
| 1. It is hard for our family to plan activities like vacations | 0 | 1 | 2 | 3 | 4 |
| 2. It is hard for our family to get enough rest | 0 | 1 | 2 | 3 | 4 |
| 3. I think money is a problem in our family | 0 | 1 | 2 | 3 | 4 |
| 4. I think our family has a lot of problems | 0 | 1 | 2 | 3 | 4 |

Table1 lengend: table 1 shows the detailed dimension of proxy-report of PedsQL ™Neuromuscular Module.

**Supplementary table2:**

**PedsQL™ Family Impact Module**

*In the past ONE month, as a result of your child’s health, how much of a problem have you had with…*

| **PHYSICAL FUNCTIONING *(problems with…)*** | **Never** | **Almost Never** | **Some- times** | **Often** | **Almost Always** |
| --- | --- | --- | --- | --- | --- |
| 1. I feel tired during the day | 0 | 1 | 2 | 3 | 4 |
| 2. I feel tired when I wake up in the morning | 0 | 1 | 2 | 3 | 4 |
| 3. I feel too tired to do the things I like to do | 0 | 1 | 2 | 3 | 4 |
| 4. I get headaches | 0 | 1 | 2 | 3 | 4 |
| 5. I feel physically weak | 0 | 1 | 2 | 3 | 4 |
| 6. I feel sick to my stomach | 0 | 1 | 2 | 3 | 4 |
| **EMOTIONAL FUNCTIONING *(problems with…)*** | **Never** | **Almost Never** | **Some- times** | **Often** | **Almost Always** |
| 1. I feel anxious | 0 | 1 | 2 | 3 | 4 |
| 2. I feel sad | 0 | 1 | 2 | 3 | 4 |
| 3. I feel angry | 0 | 1 | 2 | 3 | 4 |
| 4. I feel frustrated | 0 | 1 | 2 | 3 | 4 |
| 5. I feel helpless or hopeless | 0 | 1 | 2 | 3 | 4 |
| **SOCIAL FUNCTIONING *(problems with…)*** | **Never** | **Almost Never** | **Some- times** | **Often** | **Almost Always** |
| 1. I feel isolated from others | 0 | 1 | 2 | 3 | 4 |
| 2. I have trouble getting support from others | 0 | 1 | 2 | 3 | 4 |
| 3. It is hard to find time for social activities | 0 | 1 | 2 | 3 | 4 |
| 4. I do not have enough energy for social activities | 0 | 1 | 2 | 3 | 4 |
| **COGNITIVE FUNCTIONING *(problems with…)*** | **Never** | **Almost Never** | **Some- times** | **Often** | **Almost Always** |
| 1. It is hard for me to keep my attention on things | 0 | 1 | 2 | 3 | 4 |
| 2. It is hard for me to remember what people tell me | 0 | 1 | 2 | 3 | 4 |
| 3. It is hard for me to remember what I just heard | 0 | 1 | 2 | 3 | 4 |
| 4. It is hard for me to think quickly | 0 | 1 | 2 | 3 | 4 |
| 5. I have trouble remembering what I was just thinking | 0 | 1 | 2 | 3 | 4 |
| **COMMUNICATION *(problems with…)*** | **Never** | **Almost Never** | **Some- times** | **Often** | **Almost Always** |
| 1. I feel that others do not understand my family’s  situation | 0 | 1 | 2 | 3 | 4 |
| 2. It is hard for me to talk about my child’s health with others | 0 | 1 | 2 | 3 | 4 |
| 3. It is hard for me to tell doctors and nurses how I feel | 0 | 1 | 2 | 3 | 4 |
| **WORRY *(problems with…)*** | **Never** | **Almost Never** | **Some- times** | **Often** | **Almost Always** |
| 1. I worry about whether or not my child’s medical  treatments are working | 0 | 1 | 2 | 3 | 4 |
| 2. I worry about the side effects of my child’s medications/medical treatments | 0 | 1 | 2 | 3 | 4 |
| 3. I worry about how others will react to my child’s condition | 0 | 1 | 2 | 3 | 4 |
| 4. I worry about how my child’s illness is affecting other family members | 0 | 1 | 2 | 3 | 4 |
| 5. I worry about my child’s future | 0 | 1 | 2 | 3 | 4 |
| **DAILY ACTIVITIES *(problems with…)*** | **Never** | **Almost Never** | **Some- times** | **Often** | **Almost Always** |
| 1. Family activities taking more time and effort | 0 | 1 | 2 | 3 | 4 |
| 2. Difficulty finding time to finish household tasks | 0 | 1 | 2 | 3 | 4 |
| 3. Feeling too tired to finish household tasks | 0 | 1 | 2 | 3 | 4 |
| **FAMILY RELATIONSHIPS *(problems with…)*** | **Never** | **Almost Never** | **Some- times** | **Often** | **Almost Always** |
| 1. Lack of communication between family members | 0 | 1 | 2 | 3 | 4 |
| 2. Conflicts between family members | 0 | 1 | 2 | 3 | 4 |
| 3. Difficulty making decisions together as a family | 0 | 1 | 2 | 3 | 4 |
| 4. Difficulty solving family problems together | 0 | 1 | 2 | 3 | 4 |
| 5. Stress or tension between family members | 0 | 1 | 2 | 3 | 4 |

Table2 lengend: table 2 shows the detailed dimension of PedsQL™ Family Impact Module.
